# Adaptive Expectation–Maximization-Based Kalman Filter/Finite Impulse Response Filter for MEMS-INS-Based Posture Capture of Human Upper Limbs

**DOI:** 10.3390/mi15040440

**Published:** 2024-03-26

**Authors:** Mingxu Sun, Yichen Li, Rui Gao, Jingwen Yu, Yuan Xu

**Affiliations:** 1 School of Electrical Engineering, University of Jinan, Jinan 250022, China; cse_sunmx@ujn.edu.cn (M.S.); 202221200878@stu.ujn.edu.cn (R.G.); 202321201001@stu.ujn.edu.cn (J.Y.); 2 Jinan Key Laboratory of Rehabilitation and Evaluation of Motor Dysfunction, Jinan 250022, China; 3 Shandong University of Science and Technology, Jinan 250031, China; 202113040316@sdust.edu.cn

**Keywords:** INS, FIR, human upper limbs motion capture

## Abstract

To obtain precise positional information, in this study, we propose an adaptive expectation–maximization (EM)-based Kalman filter (KF)/finite impulse response (FIR) integrated filter for inertial navigation system (INS)-based posture capture of human upper limbs. Initially, a data fusion model for wrist and elbow position is developed. Subsequently, the *Mahalanobis* distance is utilized to evaluate the performance of the filter. The integrated filter employs the EM-based KF to enhance noise estimation accuracy when the performance of KF declines. Conversely, upon deterioration in the performance of the EM-based KF, which is evaluated using the *Mahalanobis* distance, the FIR filter is employed to maintain the effectiveness of the data fusion filter. This research utilizes the proposed EM-based KF/FIR integrated filter to ascertain wrist and elbow positions. The empirical results demonstrate the proficiency of the proposed approach in estimating these positions, thereby overcoming the challenge and highlighting its inherent effectiveness.

## 1. Introduction

Recently, the number of patients with motor function injuries has been increasing every year, posing significant challenges to their lives and their families [1,2]. Rehabilitation training is crucial for these patients to help them overcome these challenges as soon as possible. The accurate implementation of rehabilitation training has emerged as a focal research area in medical rehabilitation and training equipment domains. In the equipment domain and with advancements in science and technology, the precise acquisition of human joint positions has gradually become a new research hotspot. In particular, visual posture capture and inertial navigation system (INS)-based posture capture are the prevalent examples. For instance, ref. [3] detailed the use of motion capture sensors for acquiring human motion data, which are subsequently processed in accordance with relevant data formats. Ref. [4] reported a 3-D tracking of upper limb movement by using two inertial sensor systems. Additionally, the scheme for upper limb motion monitoring in neurorehabilitation utilizing low-cost inertial sensors such as those found in Sony Move, Nintendo Wii (Wii Remote with Wii MotionPlus), and smartphones has been developed [5]. Ref. [6] employed two wearable inertial sensors that are placed near the wrist and elbow joints to measure the human motion of the upper limbs. Research on video-recognition-based virtual reality for three-dimensional human motion pose capture, as discussed in [7,8], reported favorable results in accurately capturing and recognizing dual-category human motion gestures. Ref. [9] presented a refined technique for reconstructing accurate motion from partially captured and noisy postures using Kinect, with experiments demonstrating significant accuracy of posture recognition under severe occlusion conditions. Ref. [10] proposed a computer vision algorithm for automatic construction of a human body skeleton model, employing a method that segments the body into primary components by calculating the curvature of a B-spline parameterized human contour. This approach effectively addressed the complex issue of initialization in a vision-based markerless motion capture system for the human body. Investigations into wearable sensor methodologies for assessing lower limb motion are reported in [11,12], guiding a novel, self-contained, and universally adaptable system capable of consistent tracking of human lower limbs without substantial differences. Ref. [13] reported an innovative wearable sensor system developed utilizing a commercial system-in-package with inertial and magnetic sensors. Further, Ref. [14] reported a new algorithm for filtering foot motion and estimating foot kinematics during normal walking using inertial and magnetic sensors in relation to an earth-fixed reference frame. Lastly, Refs. [15,16] discussed a monitoring system based on inertial sensors for measuring and tracking upper limb movement in humans utilizing two wearable inertial sensors positioned close to the wrist and elbow joints.

Employing the Kalman filter (KF) effectively mitigates measurement drift, demonstrating commendable accuracy and reliability. A novel algorithm for motion tracking has been developed by fusing data from two wearable inertial sensors positioned near the wrist and elbow joints. Empirical findings showcased that the algorithm exhibited proficiency in achieving unwavering motion tracking of human arms over a 45 s duration being devoid of any perceptible measurement drifts [17]. Despite the capabilities of the aforementioned measurement methods for human motion capture, they suffer from many limitations. The INS-based method is also prone to error accumulation, and visual solutions encounter recognition challenges in scenarios in which limbs intersect. Employed with the equipment, data fusion filters have shown potential in improving localization precision [18]. A prominent example of such filters is the KF, which has been the subject of numerous fusion efforts [19]. In [20], a novel approach involving the utilization of a predictive quaternion KF is reported for continuous wireless tracking of lower limb posture of humans, effectively overcoming wireless communication outages. In addition, Ref. [21] reported a robust KF by deriving robust estimators for Kalman filtering that incorporate constraints on state parameters by leveraging the principles of the generalized maximum likelihood Lagrangian condition. Simulation results and semiphysical trials revealed the efficacy of an adaptive KF in improving in the accuracy of state variable estimation. Ref. [22] introduced a novel expectation–maximization (EM) algorithm with guaranteed convergence to derive the maximum likelihood estimator (MLE) solution. Furthermore, Ref. [23] discussed the sigma-point update of a cubature KF of the Global Navigation Satellite System (GNSS)/INS integrated environment. Notably, the discussed KF-based methods require an accurate data fusion model and a comprehensive noise description, which is hard to achieve in practice [24].

To surmount this obstacle, the implementation of a finite impulse response (FIR) filter is proposed. In [25], an improved FIR filter was proposed for ultrawide-band (UWB) localization, integrating the FIR filter with a predictive model and extreme learning machine (ELM) to enhance the accuracy of UWB-based localization. Ref. [26] introduced an improved iterative FIR state estimator. Although the FIR filter showed increased robustness, its localization accuracy may not surpass that of KF when the KF model is precise. The increasing prevalence of motor function injuries significantly impacts the lives of patients and their families. Thus, accurate implementation of rehabilitation training for patients has become increasingly central in research in this field. This study introduces an adaptive EM-based KF/FIR integrated filter for INS-based posture capture of human upper limbs. Initially, a data fusion model for the wrist and elbow positions is developed. The *Mahalanobis* distance is then employed to assess the performance of the filter. In the integrated filter, when the performance of KF deteriorates, the EM-based KF is utilized to improve the noise estimation accuracy. Subsequently, the *Mahalanobis* distance is used to evaluate the performance of the EM-based KF. Upon further decline in the performance of the EM-based KF, the FIR filter is employed to maintain the effectiveness of the data fusion filter. This research employs the proposed EM-based KF/FIR integrated filter for measuring the wrist and elbow positions. Empirical results demonstrate the effectiveness of the method in providing accurate position estimations of its capacity to overcome the challenge. This study contributes significantly in the following areas:

- An INS-based motion model for human upper limbs is formulated, focusing on the wrist and elbow positions. The state vector comprises their position and velocity in the East–North–Up frame. Further, IMU-measured positions are employed as the input. The output of the two data fusion filters are used to determine the posture of human upper limbs.
- A EM-based KF/FIR integrated filtering method is designed. It leverages the INS-based motion model of human upper limbs, using KF to estimate wrist and elbow positions from INS-based measurements. The *Mahalanobis* distance is used to evaluate the performance of the filter, employing the EM-based method and subsequently the FIR filter as the performance of KF deteriorates.
- Experimental results affirm the superior performance of the proposed algorithms compared to traditional counterparts. A real-world test using two IMUs for INS-based wrist and elbow position measurements and Kinect 2.0 used to provide reference values demonstrate the effectiveness of the proposed EM-based KF/FIR integrated filter over traditional KF and FIR filters.

The remaining sections of this paper are organized as follows: Section 2 delves into posture capture of human upper limbs based on INS. Section 3 details the design of the EM-based KF/FIR filter used for capturing motion of human upper limbs. Section 4 summarizes experimental tests, and conclusions are presented in Section 5.

## 2. INS-Based Posture Capture of Human Upper Limbs

**Figure 1:**
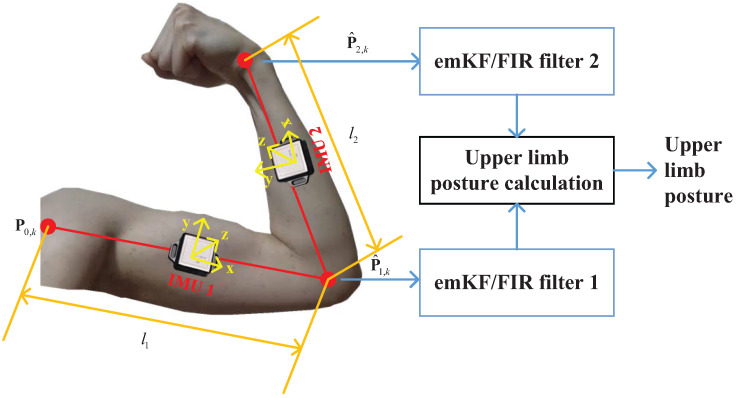
INS-based posture capture of human upper limbs using the EM-based KF/FIR filter.

This section outlines the model design for capturing human upper limb motion using an INS-based posture capture scheme, as depicted in Figure 1. As seen in the figure, two IMUs are affixed between the joints to measure the attitudes of the humerus and radius using accelerometer and gyroscope data from the devices. Initially, the shoulder’s position $P_{0,k}$ at the time index *k* is obtained. The IMU then computes the attitude transfer matrix $T_{0,k}^{1}$ from $P_{0,k}$ to the elbow position $P_{1,k}$, which is calculated using the following equation:

(1) $${\mathrm{Po}}_{1,k}={\mathrm{Po}}_{0,k}+l_{1}T_{0,k}^{1}\;,$$

where $T_{0,k}^{1}=T_{n,k}^{1}T_{0,k}^{n}$. Employing ${\mathrm{Po}}_{1,k}$ and $T_{1,k}^{2}$ measured by IMU 2, the wrist position ${\mathrm{Po}}_{2,k}$ is computed as follows:

(2) $${\mathrm{Po}}_{2,k}={\mathrm{Po}}_{1,k}+l_{2}T_{1,k}^{2}\;,$$

where $T_{1,k}^{2}=T_{n,k}^{2}T_{1,k}^{n}$. In this study, the measurements ${\hat{P}o}_{1,k}$ and ${\hat{P}o}_{2,k}$ were utilized by the EM-based KF/FIR filters 1 and 2, respectively, the design of which is elucidated in the subsequent section. The outputs from these filters are input to the motion capture calculations for upper limbs.

## 3. EM-Based KF/FIR Filter for Position Estimation

In this section, we articulate the method for position estimation based on the EM-based KF/FIR filter, as illustrated in Figure 1. Initially, the data fusion model is discussed. This is followed by a brief introduction of the EM-based KF and FIR filters. Finally, the principle of the EM-based KF/FIR filter is expounded for INS-based posture capture.

### 3.1. Data Fusion Model

Based on the scheme shown in Figure 1, a dual-data-fusion model is requisite for the dual-data-fusion filter. The state equation for the ith EM-based KF/FIR filter is expressed as follows:

(3) $$\underset{L_{k}^{i-}}{\underbrace{\left[\begin{array}{c} x_{k}^{i-}\\ {\mathrm{vx}}_{k}^{i-}\\ y_{k}^{i-}\\ {\mathrm{vy}}_{k}^{i-}\\ z_{k}^{i-}\\ vz_{k}^{i-} \end{array}\right]}}=\underset{T}{\underbrace{\left[\begin{array}{cccccc} 1 & \Delta k & 0 & 0 & 0 & 0\\ 0 & 1 & 0 & 0 & 0 & 0\\ 0 & 0 & 1 & \Delta k & 0 & 0\\ 0 & 0 & 0 & 1 & 0 & 0\\ 0 & 0 & 0 & 0 & 1 & \Delta k\\ 0 & 0 & 0 & 0 & 0 & 1 \end{array}\right]}}\underset{L_{k}^{i}}{\underbrace{\left[\begin{array}{c} x_{k}^{i}\\ {\mathrm{vx}}_{k}^{i}\\ y_{k}^{i}\\ {\mathrm{vy}}_{k}^{i}\\ z_{k}^{i}\\ vz_{k}^{i} \end{array}\right]}}+w_{k}^{i}\;,$$

where *i* denotes the elbow (i=1) and wrist (i=2), ${\mathrm{Po}}_{i,k}={\left(x_{k}^{i},y_{k}^{i},z_{k}^{i}\right)}^{T}$ is the elbow’s or wrist’s position, ${\mathrm{Vel}}_{i,k}={\left({\mathrm{vx}}_{k}^{i},{\mathrm{vy}}_{k}^{i},{\mathrm{vz}}_{k}^{i}\right)}^{T}$ is the elbow’s or wrist’s velocity, Δk is the sampling time, and $w_{k}^{i}∼N\left(0,Q\right)$ is the system noise.

(4) $${\mathrm{Po}}_{i,k}=\underset{H}{\underbrace{\left[\begin{array}{cccccc} 1 & 0 & 0 & 0 & 0 & 0\\ 0 & 0 & 1 & 0 & 0 & 0\\ 0 & 0 & 0 & 0 & 1 & 0 \end{array}\right]}}L_{k}^{i-}+v_{k}^{i}\;,$$

where $v_{k}^{i}∼N\left(0,R\right)$ is the measurement noise.

### 3.2. EM-Based KF

Based on the model (1) and (2), the kF can be calculated using the following equations: First, one-step prediction is performed.

(5) $$L_{k}^{i-}={\mathrm{TL}}_{k}^{i}+w_{k}^{i}\;,$$

(6) $$P_{k}^{i-}={\mathrm{TP}}_{k}^{i}T^{T}+Q\;,$$

Then, with the measurement $P_{i,k}$, KF employs the following equations:

(7) $$K_{k}^{i}=P_{k}^{i-}H^{T}{\left({\mathrm{HP}}_{k}^{i-}H^{T}+R\right)}^{-1}\;,$$

(8) $$L_{k}^{i}=L_{k}^{i-}+K_{k}^{i}\left({\mathrm{Po}}_{i,k}-{\mathrm{HL}}_{k}^{i-}\right)\;,$$

(9) $$P_{k}^{i}=\left(I-K_{k}^{i}H\right)P_{k}^{i-}{\left(I-K_{k}^{i}H\right)}^{T}+K_{k}^{i}R{\left(K_{k}^{i}\right)}^{T}\;,$$

The KF algorithm based on the model (1) and (2) is can be found in Algorithm 1 below:
**Algorithm 1:** KF method for the model (1) and (2)
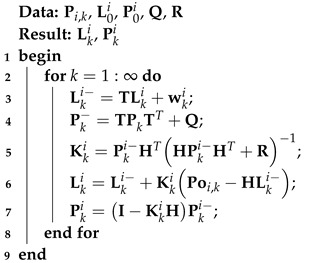


Note that the KF method depends on the accuracy of the model, which is hard to achieve in practice. To enhance the robustness of data fusion methods, the EM-based KF has been proposed [27]. This method employs the joint log-likelihood function $g_{\alpha_{k}}\left(L_{k}^{i},Po_{i,1:k-1}\right)\overset{\Delta }{=}logp_{\alpha_{k}}\left(L_{k}^{i},Po_{i,1:k-1}\right)$ as the minimum variance estimate $f\left(\alpha_{k},\alpha_{k}^{\left(l\right)}\right)$:

(10) $$\begin{array}{c} g_{\alpha_{k}}\left(L_{k}^{i},Po_{i,1:k-1}\right)\approx f\left(\alpha_{k},\alpha_{k}^{\left(l\right)}\right)\overset{\Delta }{=}E\left(g_{\alpha_{k}}\left(L_{k}^{i},Po_{i,1:k-1}\right)|\alpha_{k}^{\left(l\right)},Po_{i,1:k}\right)\\ \;\;\;\;\;\;\;\;\;\;\;\;\;\;\;\;\;\;\;\;\;\;\;=\int logp_{\alpha_{k}}\left(L_{k}^{i},Po_{i,1:k}\right)p_{\alpha_{k}^{\left(l\right)}}\left(L_{k}^{i}|Po_{i,1:k}\right)dL_{k}^{i} \end{array}\;,$$

This EM-based KF method involves an expectation step (E-step) and a maximization step (M-step). For the E-step, we can obtain the following equation:

(11) $$\begin{array}{c} p_{\alpha_{k}}\left(L_{k}^{i},Po_{i,1:k}\right)=p_{\alpha_{k}}\left(Po_{i,k}|L_{k}^{i},Po_{i,1:k}\right)p_{\alpha_{k}}\left(L_{k}^{i}|Po_{i,1:k-1}\right)p\left(Po_{i,1:k-1}\right)\\ \;\;\;\;\;\;\;\;\;\;\;\;\;\;\;\;\;\;\;\;\;\;\;\;\;=p_{\alpha_{k}}\left(Po_{i,k}|L_{k}^{i}\right)p_{\alpha_{k}}\left(L_{k}^{i}|Po_{i,1:k-1}\right)p\left(Po_{i,1:k-1}\right) \end{array}\;,$$

Based on the KF method presented as Algorithm 1, we can obtain

(12) $$p_{\alpha_{k}}\left(L_{k}^{i}|Po_{i,1:k-1}\right)=N\left(L_{k}^{i-},P_{k}^{i-}\right)\;,$$

(13) $$p_{\alpha_{k}}\left(Po_{i,k}|Po_{i,1:k-1}\right)=N\left({\mathrm{HL}}_{k}^{i-},R_{k}\right)\;,$$

Employing Equations (11)–(13), we obtain the joint log-likelihood function as follows:

(14) $$\begin{array}{c} \log p_{\alpha_{k}}\left(L_{k}^{i},Po_{i,1:k-1}\right)=-\frac{1}{2}\log\left|R_{k}\right|-\frac{1}{2}\left({\mathrm{Po}}_{i,k}-{\mathrm{HL}}_{k}^{i-}\right)R_{k}^{-1}{\left({\mathrm{Po}}_{i,k}-{\mathrm{HL}}_{k}^{i-}\right)}^{T}\\ \;\;\;\;\;\;\;\;\;\;\;\;\;\;\;\;\;\;\;\;\;\;\;\;\;\;\;-\frac{1}{2}{\left(L_{k}^{i}-L_{k}^{i-}\right)}^{T}{\left(P_{k}^{i-}\right)}^{-1}\left(L_{k}^{i}-L_{k}^{i-}\right)+O_{\alpha_{k}}\;\; \end{array}\;,$$

where the $O_{\alpha_{k}}$ denotes a constant. Here, we can compute the posterior probability density function (PDF) $p_{\alpha_{k}^{\left(l\right)}}\left(L_{k}^{i}|Po_{i,1:k}\right)$ via a small $l^{th}$ step iteration of $L_{k}^{i(l)}$ and $P_{k}^{i(l)}$. Thus, we can determine that

(15) $$p_{\alpha_{k}^{\left(l\right)}}\left(L_{k}^{i}|Po_{i,1:k}\right)=N\left(L_{k}^{i\left(l\right)},P_{k}^{i\left(l\right)}\right)\;,$$

Finally, substituting (15) in (10), we obtain:

(16) $$f\left(\alpha_{k},\alpha_{k}^{\left(l\right)}\right)=-\frac{1}{2}\log\left|R_{k}\right|-\frac{1}{2}tr\left(\Omega_{1,k}R_{k}^{-1}\right)-\frac{1}{2}\log\left|P_{k}^{i-}\right|-\frac{1}{2}tr\left(\Omega_{2,k}{\left(P_{k}^{i-}\right)}^{-1}\right)+O_{\alpha_{k}}\;\;,$$

Here,

(17) $$\begin{array}{c} \Omega_{1,k}=\int \left(Po_{i,k}-{\mathrm{HL}}_{k}^{i\left(l\right)}\right){\left(Po_{i,k}-{\mathrm{HL}}_{k}^{i\left(l\right)}\right)}^{T}N\left(L_{k}^{i\left(l\right)},P_{k}^{i\left(l\right)}\right)dL_{k}^{i}\\ \;\;\;\;\;\;\;=\left(Po_{i,k}-{\mathrm{HL}}_{k}^{i-\left(l+1\right)}\right){\left(Po_{i,k}-{\mathrm{HL}}_{k}^{i-\left(l+1\right)}\right)}^{T}+{\mathrm{HP}}_{k}^{i\left(l+1\right)}H^{T} \end{array}\;,$$

(18) $$\begin{array}{c} \Omega_{2,k}=\int {\left(L_{k}^{i}-L_{k}^{i-}\right)\left(L_{k}^{i}-L_{k}^{i-}\right)}^{T}N\left(L_{k}^{i\left(l\right)},P_{k}^{i\left(l\right)}\right)dL_{k}^{i}=P_{k}^{i\left(l\right)}+\left(L_{k}^{i\left(l\right)}-L_{k}^{i-}\right){\left(L_{k}^{i\left(l\right)}-L_{k}^{i-}\right)}^{T} \end{array}\;,$$

To the M-step, we can compute the following equation.

(19) $$\frac{\partial f\left(\alpha_{k},\alpha_{k}^{\left(l\right)}\right)}{\partial P_{k}^{i-}}=0,\frac{\partial f\left(\alpha_{k},\alpha_{k}^{\left(l\right)}\right)}{\partial R_{k}^{i}}=0\;,$$

From (19), we obtain

(20) $$\frac{\partial f\left(\alpha_{k},\alpha_{k}^{\left(l\right)}\right)}{\partial P_{k}^{i-}}=-\frac{1}{2}{\left(P_{k}^{i-\left(l+1\right)}\right)}^{-1}+\frac{1}{2}{\left(P_{k}^{i-\left(l+1\right)}\right)}^{-1}\Omega_{1,k}{\left(P_{k}^{i-\left(l+1\right)}\right)}^{-1}=0\;,$$

(21) $$\frac{\partial f\left(\alpha_{k},\alpha_{k}^{\left(l\right)}\right)}{\partial R_{k}^{i}}=-\frac{1}{2}{\left(R_{k}^{i\left(l+1\right)}\right)}^{-1}+\frac{1}{2}{\left(R_{k}^{i\left(l+1\right)}\right)}^{-1}\Omega_{2,k}{\left(R_{k}^{i\left(l+1\right)}\right)}^{-1}=0\;,$$

Thus, we obtain

(22) $$\Omega_{1,k}=P_{k}^{i-\left(l+1\right)}\;,$$

(23) $$\Omega_{2,k}=R_{k}^{i\left(l+1\right)}\;,$$

Thus, we obtain Algorithm 2:
**Algorithm 2:** EM-based KF method for the model (1) and (2)
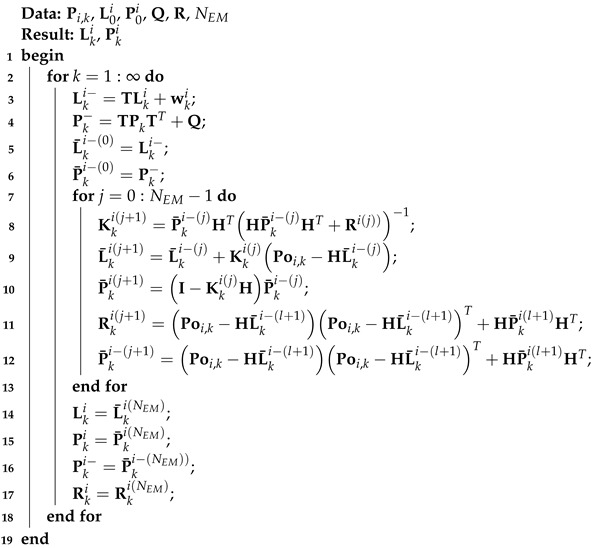


### 3.3. FIR Filter

From Algorithm 1, we see that the capability of KF to provide estimations is contingent upon the accuracy of the underlying model, which is often difficult to achieve. To this end, a solution involving the FIR filter has been proposed in [28]. In this study, based on the models (1) and (2), we perform data fusion using the FIR filter and recent measurements from the time index $k-N_{FIR}+1$ to *k*. Here, $N_{FIR}$ denotes the filtering window size, and *j* indicates the iteration number of the FIR filter. This one-step prediction of the FIR filter can be computed by the following equation:

(24) $$L_{j}^{i-}={\mathrm{TL}}_{j}^{i}\;,$$

(25) $$P_{j}^{i-}={\mathrm{TP}}_{j}^{i}T^{T}+Q\;,$$

Thereafter, the measurement update can be performed as follows:

(26) $$K_{j}^{i}={\left(HH^{T}+{\left(\mathrm{TH}T^{T}\right)}^{-1}\right)}^{-1}H\;,$$

(27) $$L_{j}^{i}=L_{j}^{i-}+K_{j}^{i}\left(Po_{i,j}-{\mathrm{HL}}_{j}^{i-}\right)\;,$$

(28) $$P_{j}^{i}=\left(I-K_{j}^{i}H\right)P_{j}^{i-}{\left(I-K_{j}^{i}H\right)}^{T}+K_{j}^{i}R{\left(K_{j}^{i}\right)}^{T}\;,$$

Thus, we obtain the FIR method for the models (1) and (2) presented as Algorithm 3.
**Algorithm 3:** FIR method for the model (1) and (2)
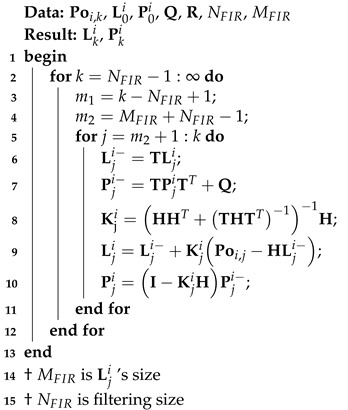


### 3.4. EM-Based Kf/FIR Integrated Filter

This section outlines the design of the EM-based KF/FIR integrated filter, incorporating the previously mentioned EM-based KF and FIR filters. The performance of the subfilters in this study is evaluated using the *Mahalanobis* distance. In the operation of the proposed EM-based KF/FIR integrated filter, the initial step involves one-step prediction by KF, as expressed in Equations (5) and (6). Subsequently, the *Mahalanobis* distance is computed as per the following equation:

(29) $$D_{k}={\left(Po_{i,k}-{\mathrm{HL}}_{k}^{i-}\right)}^{T}R_{k}^{-1}\left(Po_{i,k}-{\mathrm{HL}}_{k}^{i-}\right)\;,$$

**Figure 2:**
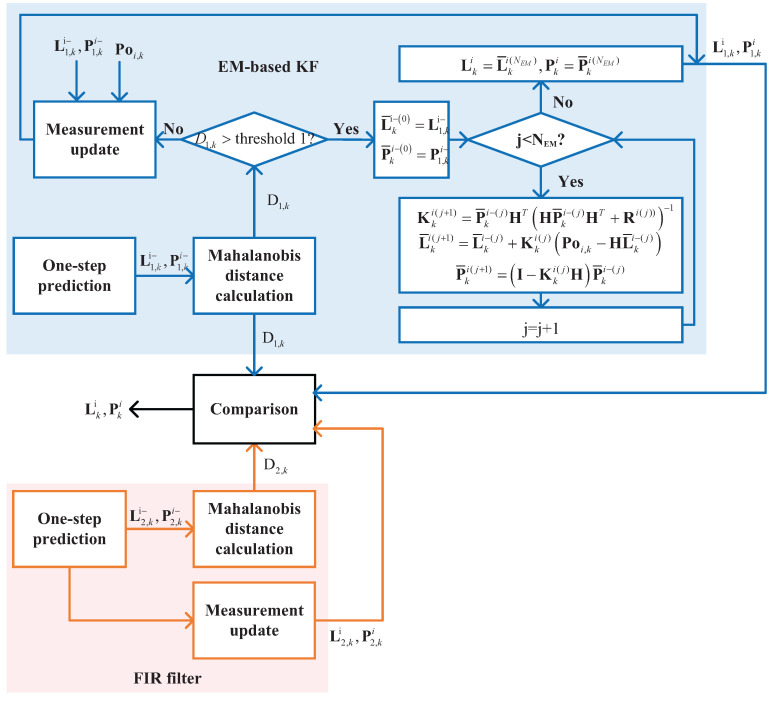
The structure of the EM-based Kf/FIR integrated filter.

If $D_{k}<threshold1$, the measurement is updated via Equations (7)–(9). Otherwise, we first set ${\bar{L}}_{k}^{i-\left(0\right)}=L_{k}^{i-}$ and ${\bar{P}}_{k}^{i-\left(0\right)}=P_{k}^{i-}$ and then perform the iterations listed in lines 8–12 in Algorithm 2. The second *Mahalanobis* distance is then computed using Equation (29). If $D_{k}<threshold2$, the EM-based KF operates normally; if $D_{k}>threshold2$, the FIR filter is directly used. The structure of the EM-based KF/FIR integrated filter is depicted in Figure 2.

## 4. Discussion

In this section, a real test conducted to verify the performance of the proposed method is discussed. The setting of the real test is introduced as follows.

### 4.1. Setting of the Real Test

**Figure 3:**
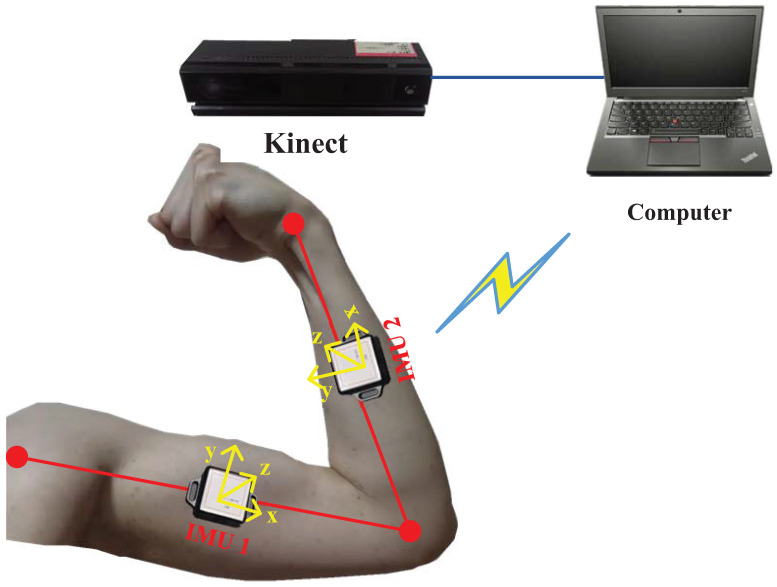
Structure of testbed used in the test.

**Figure 4:**
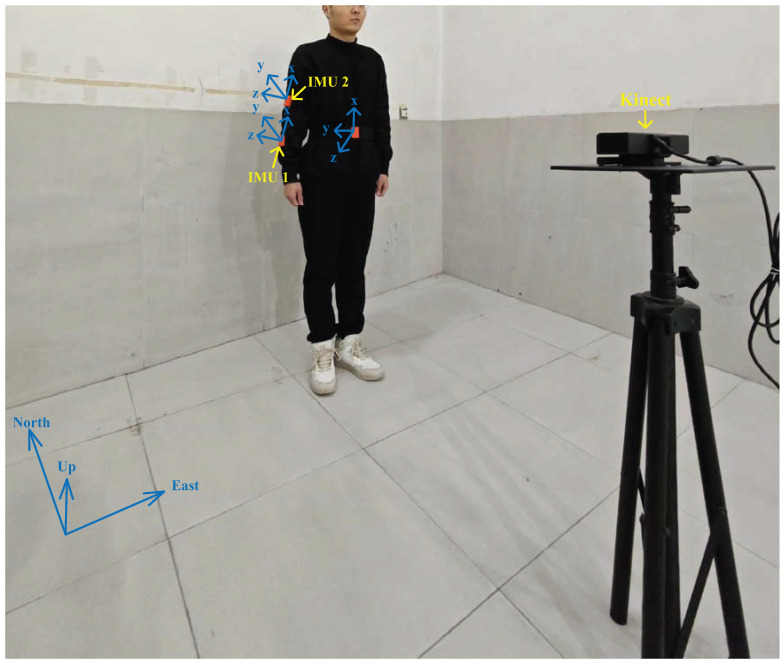
Actual experimental scenario.

In this study, focusing on human upper limbs, only two IMUs are used, which are fixed on a human upper limb. Kinect 2.0 is used to provide the reference value for vision measurement. Figure 3 illustrates the configuration of the testbed employed in this study. Practical tests were conducted in the No. 1 teaching building of the University of Jinan, with experimental scenarios displayed in Figure 4. The IMUs are affixed to the human subject and their data are transmitted wirelessly, with parameters listed in Table 1, as utilized in [29]. Unlike [29], Kinect 2.0 is used to obtain reference values, with parameters listed in Table 2 also referenced in [29]. Data acquisition from all active sensors is systematically performed using a Lenovo Legion Y9000K2020H computer, with the specifications shown in Table 3. The INS calculates the navigation results by integrating the measurement values of the inertial sensor. Therefore, the accuracy of initial alignment has a significant impact on the accuracy of INS solution. To the initial position of the shoulder, in this test, we firstly measure the position of the target human’s shoulder.

### 4.2. Positioning of the Elbow

The performance of the proposed EM-based KF/FIR integrated filter is discussed in this section. For elbow localization, we employed two real-world tests, the filter settings for the first test were as follows:

(30) $$Q=\left[\begin{array}{cccccc} \frac{\Delta k^{2}}{4} & \frac{\Delta k}{2} & 0 & 0 & 0 & 0\\ \frac{\Delta k}{2} & 1 & 0 & 0 & 0 & 0\\ 0 & 0 & \frac{\Delta k^{2}}{4} & \frac{\Delta k}{2} & 0 & 0\\ 0 & 0 & \frac{\Delta k}{2} & 1 & 0 & 0\\ 0 & 0 & 0 & 0 & \frac{\Delta k^{2}}{4} & \frac{\Delta k}{2}\\ 0 & 0 & 0 & 0 & \frac{\Delta k}{2} & 1 \end{array}\right]\;,$$

(31) $$R=\left[\begin{array}{ccc} 0.01 & 0 & 0\\ 0 & 0.01 & 0\\ 0 & 0 & 0.01 \end{array}\right]\;,$$

**Figure 5:**
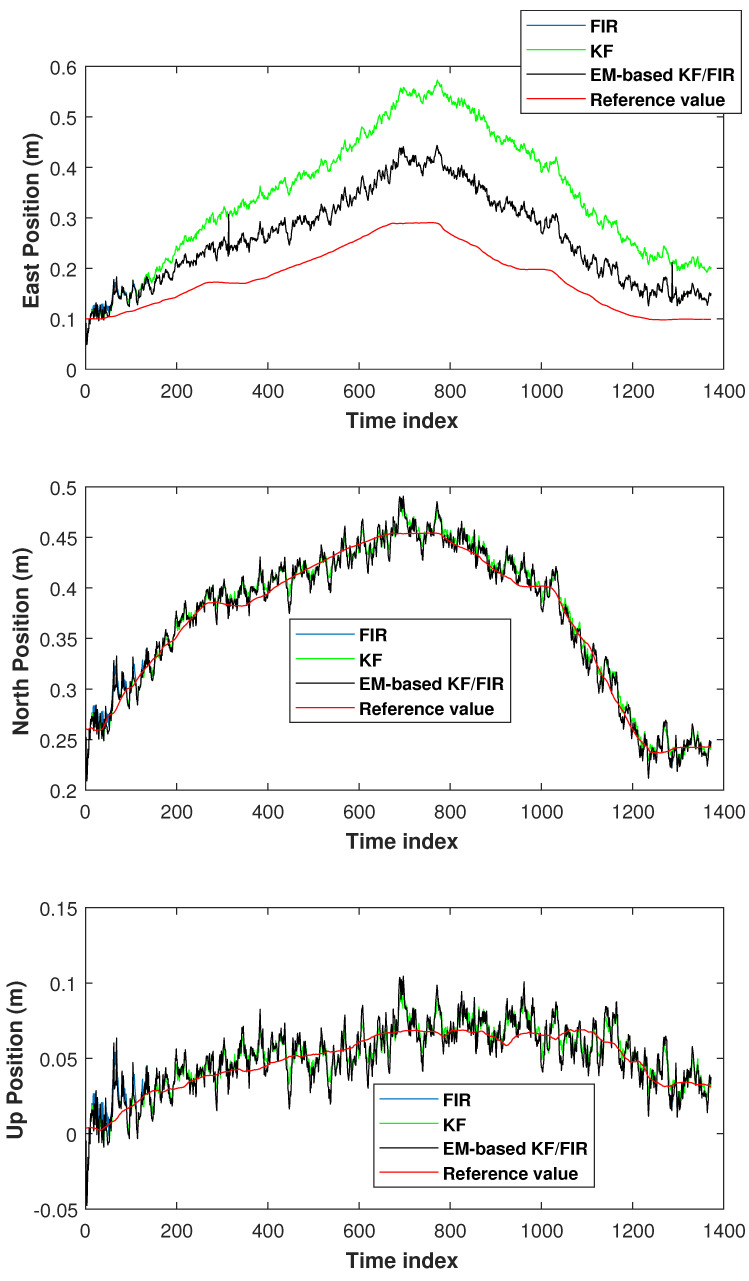
Elbow positions measured by the KF, FIR filter, and EM-based KF/FIR filter in test 1.

We set Δk=1/30 s, threshold1=2. Based on the models (1) and (2),we obtained $M_{FIR}=6$ in this test. The elbow positions measured by the KF, FIR filter, and EM-based KF/FIR filter are depicted in Figure 5. In this figure, the KF solution is represented by a green line, the FIR solution by a blue line, the proposed EM-based KF/FIR integrated filter solution by a red line, and the reference values by a red line. All solutions are close to the reference values. Notably, the FIR solution is markedly higher than the reference value in the east direction. Meanwhile, the KF solution is close to the reference values. Compared to the KF and FIR filter solutions, the EM-based KF/FIR filter solution demonstrates consistent convergence toward the benchmark value.

**Figure 6:**
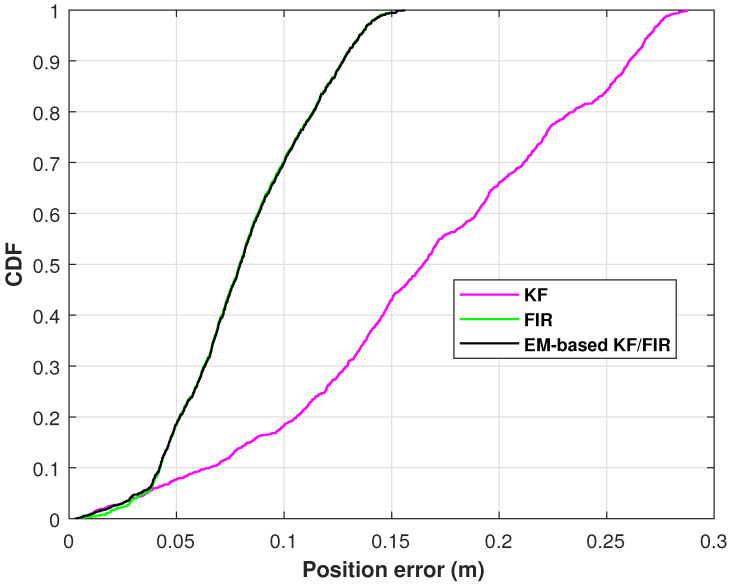
The position-error CDFs of the elbow measured by the KF, FIR filter, and EM-based KF/FIR filter in test 1.

The position-error cumulative distribution function (CDF) of the elbow measured by the KF, FIR filter, and EM-based KF/FIR filter in test 1 are shown in Figure 6. In this figure, the KF solution has the biggest position error at 0.9; the FIR and the proposed method’s solutions are similar.

The RMSEs of the KF, FIR filter, and EM-based KF/FIR filter concerning elbow positions in test 1 are listed in Table 4. We see that the mean position error is close to the KF, which has a small localization error.

The filter settings for the second test were changed as follows:

(32) $$Q=\left[\begin{array}{cccccc} \frac{\Delta k^{2}}{4} & \frac{\Delta k}{2} & 0 & 0 & 0 & 0\\ \frac{\Delta k}{2} & 1 & 0 & 0 & 0 & 0\\ 0 & 0 & \frac{\Delta k^{2}}{4} & \frac{\Delta k}{2} & 0 & 0\\ 0 & 0 & \frac{\Delta k}{2} & 1 & 0 & 0\\ 0 & 0 & 0 & 0 & \frac{\Delta k^{2}}{4} & \frac{\Delta k}{2}\\ 0 & 0 & 0 & 0 & \frac{\Delta k}{2} & 1 \end{array}\right]\times \left(0.6\times 0.3\right)\;,$$

(33) $$R=\left[\begin{array}{ccc} 0.02 & 0 & 0\\ 0 & 0.02 & 0\\ 0 & 0 & 0.02 \end{array}\right]\;,$$

**Figure 7:**
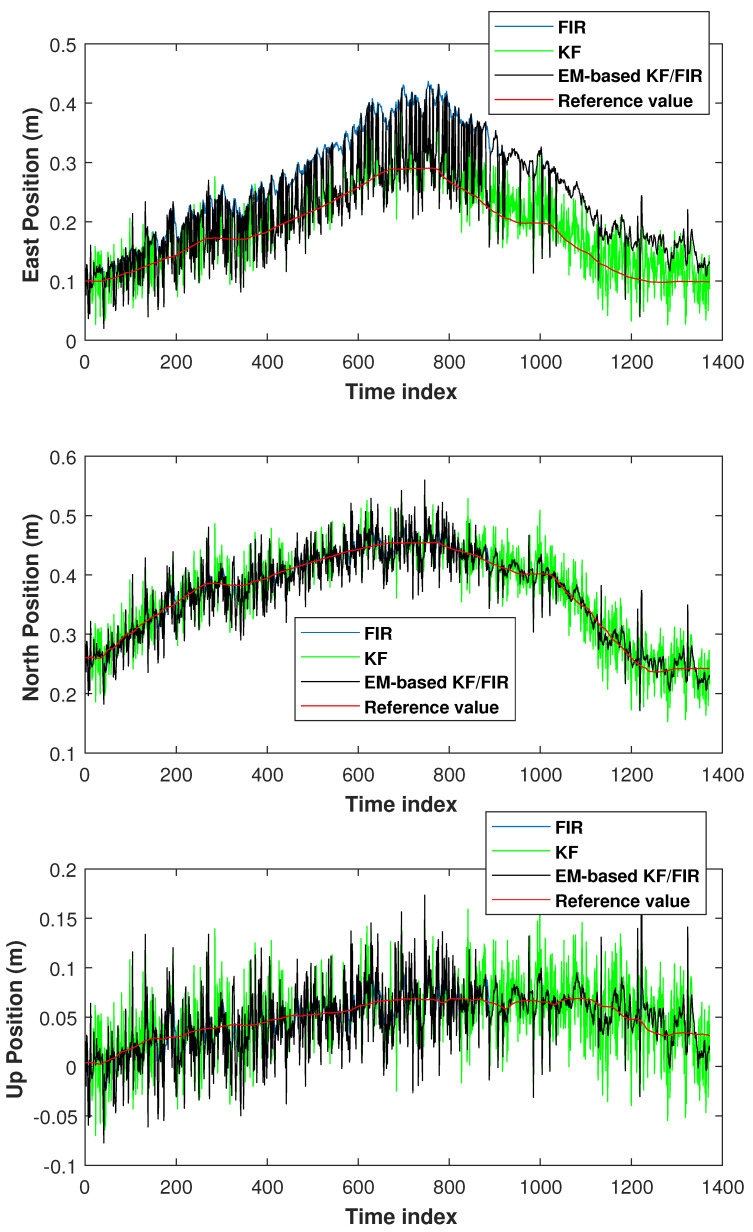
Elbow positions measured by the KF, FIR filter, and EM-based KF/FIR filter in test 2.

The elbow positions in test 2 measured by the KF, FIR filter, and EM-based KF/FIR filter are illustrated in Figure 7. All solutions are close to the reference values. From the time index 1000 to 1300, the FIR solution is noticeably higher than the reference values. The solution provided by the proposed EM-based KF/FIR filter consistently falls between the KF and FIR solutions.

**Figure 8:**
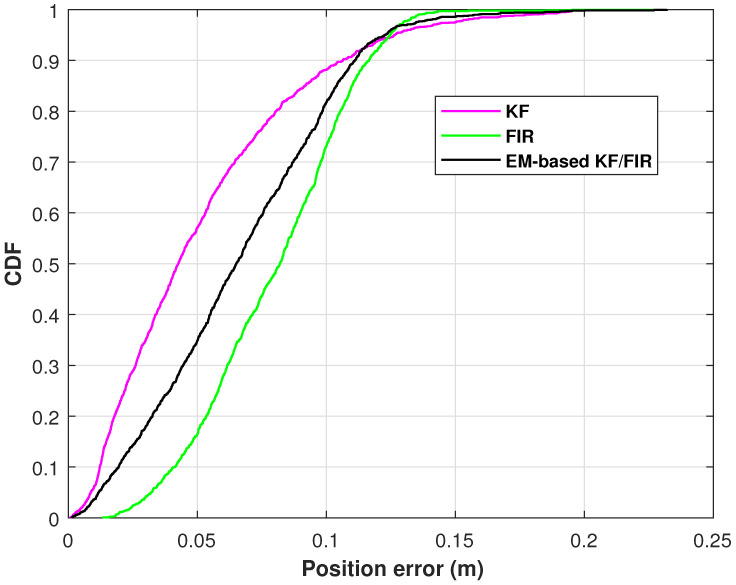
The position-error CDFs of the elbow measured by the KF, FIR filter, and EM-based KF/FIR filter in test 2.

Figure 8 illustrates the position-error CDFs of the elbow measured by the KF, FIR filter, EM-based KF/FIR filter in test 2. From the figure, the KF solution get the smallest position error, the FIR solution has the biggest position error. In this test, the solution provided by the proposed EM-based KF/FIR filter consistently falls between the KF and FIR solutions.

### 4.3. Wrist Positioning

This section focuses on the performance of the proposed EM-based KF/FIR integrated filter concerning wrist positioning. In this subsection, we also employed two tests. The filter settings for wrist localization in test 1 are as follows:

(34) $$Q=\left[\begin{array}{cccccc} \frac{\Delta k^{2}}{4} & \frac{\Delta k}{2} & 0 & 0 & 0 & 0\\ \frac{\Delta k}{2} & 1 & 0 & 0 & 0 & 0\\ 0 & 0 & \frac{\Delta k^{2}}{4} & \frac{\Delta k}{2} & 0 & 0\\ 0 & 0 & \frac{\Delta k}{2} & 1 & 0 & 0\\ 0 & 0 & 0 & 0 & \frac{\Delta k^{2}}{4} & \frac{\Delta k}{2}\\ 0 & 0 & 0 & 0 & \frac{\Delta k}{2} & 1 \end{array}\right]\times 10^{-1}\;,$$

(35) $$R=\left[\begin{array}{ccc} 0.1 & 0 & 0\\ 0 & 0.1 & 0\\ 0 & 0 & 0.1 \end{array}\right]\;,$$

**Figure 9:**
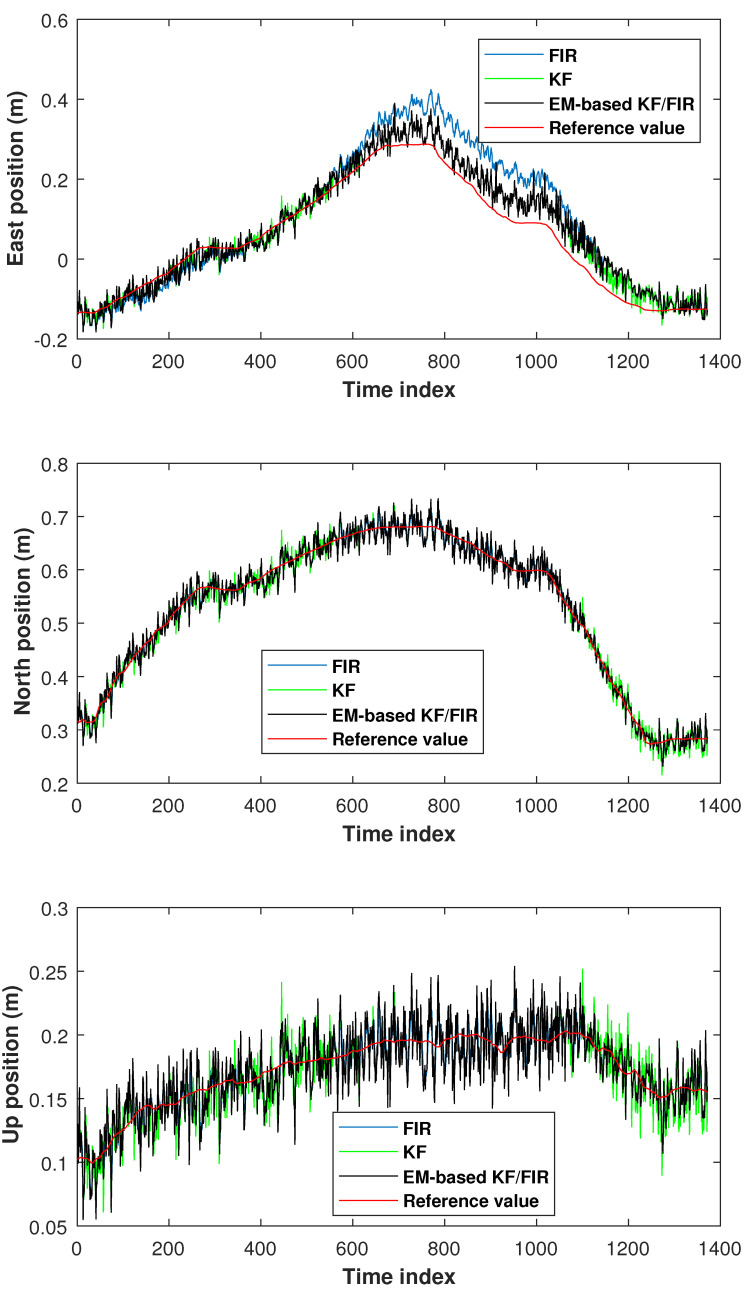
Wrist positions measured by the KF, FIR filter, and EM-based KF/FIR filter in test 1.

We set Δk=1/30 s, threshold1=2. Based on the models (1) and (2), we obtained $M_{FIR}=6$ in this test. The wrist positions measured by the KF, FIR filter, and EM-based KF/FIR filter in test 1 are displayed in Figure 9. The KF solution is represented by a green line, the FIR solution by a blue line, the proposed EM-based KF/FIR integrated filter solution by a black line, and the reference values by a red line. In the east direction, the FIR filter has biggest error from the time index 600 to 1000. During this period, the proposed EM-based KF/FIR filter and the KF filter show better performance. All solutions are close to the reference values in the north and up directions. However, the performance of KF is subpar from the time index 1100 to 1400 in these directions, while the solution provided by the proposed EM-based KF/FIR filter more consistently converges toward the reference values.

**Figure 10:**
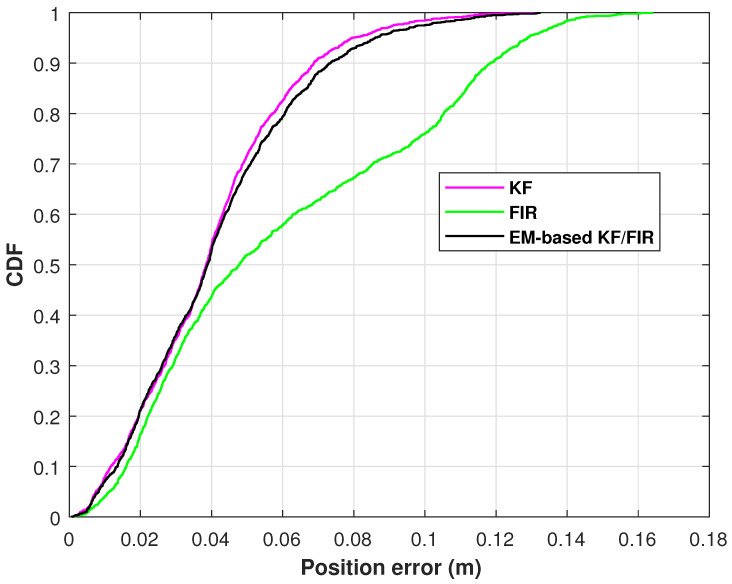
The position-error CDFs of the wrist measured by the KF, FIR filter, and EM-based KF/FIR filter in test 1.

The position-error CDF of the wrist measured by the KF, FIR filter, and EM-based KF/FIR filter in test 1 are shown in Figure 10. In this figure, the KF outperforms FIR filter. Further, the proposed EM-based KF/FIR filter solution more closely approximates the KF solution. Table 5 tabulates the RMSEs of the KF, FIR filter, and EM-based KF/FIR filter concerning wrist positions in test 1. The table reveals that the method proposed in this study achieves the smallest localization error compared to the KF and FIR filter, with its localization error value being marginally lower than that of the FIR filter.

Then, we performed the test 2 with the following settings:

(36) $$Q=\left[\begin{array}{cccccc} \frac{\Delta k^{2}}{4} & \frac{\Delta k}{2} & 0 & 0 & 0 & 0\\ \frac{\Delta k}{2} & 1 & 0 & 0 & 0 & 0\\ 0 & 0 & \frac{\Delta k^{2}}{4} & \frac{\Delta k}{2} & 0 & 0\\ 0 & 0 & \frac{\Delta k}{2} & 1 & 0 & 0\\ 0 & 0 & 0 & 0 & \frac{\Delta k^{2}}{4} & \frac{\Delta k}{2}\\ 0 & 0 & 0 & 0 & \frac{\Delta k}{2} & 1 \end{array}\right]\times 10^{-4}\;,$$

(37) $$R=\left[\begin{array}{ccc} 0.02 & 0 & 0\\ 0 & 0.02 & 0\\ 0 & 0 & 0.02 \end{array}\right]\;,$$

**Figure 11:**
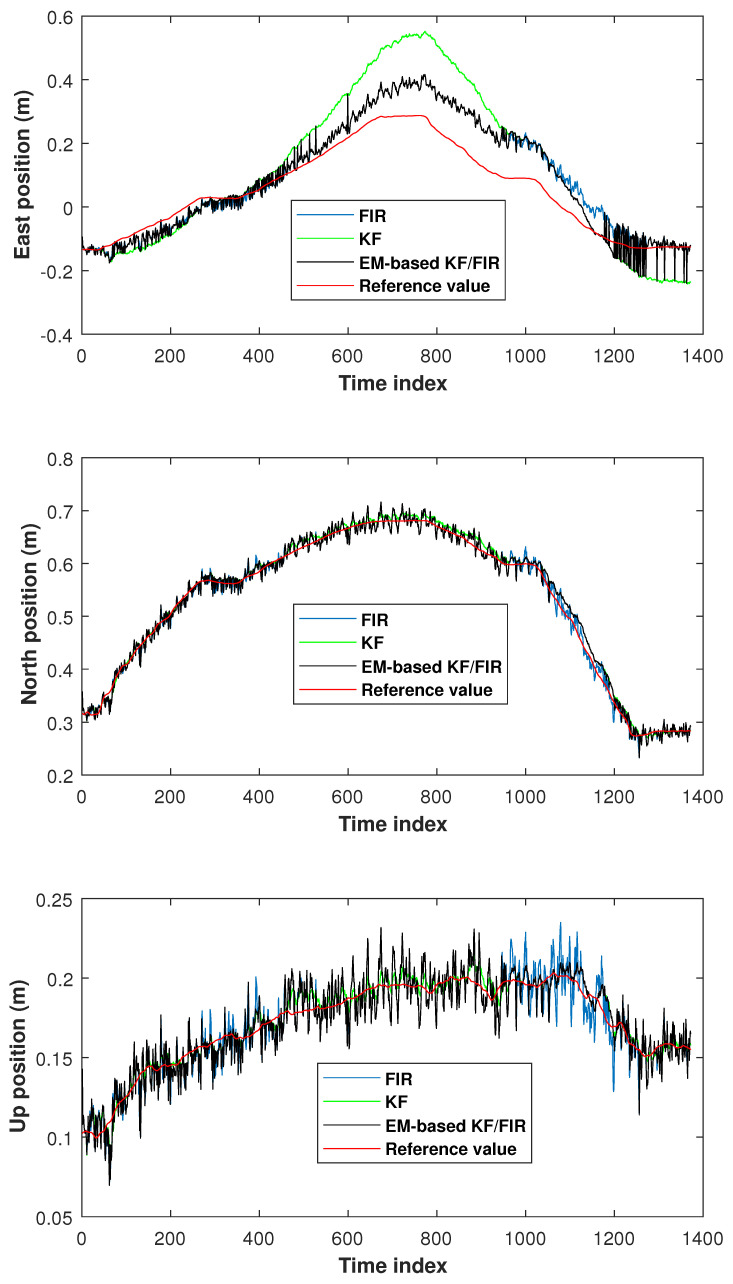
Wrist positions measured by the KF, FIR filter, and EM-based KF/FIR filter in test 2.

The wrist positions as measured by the KF, FIR filter, and EM-based KF/FIR filter in test 2 are depicted in Figure 11. In this figure, it can be seen that the KF has biggest position error in the east direction when compared with the KF and FIR filter from the time index 500 to 1000. The proposed method’s solution are closer to the reference value. In the north and up directions, the KF and the proposed method’s performances are similar.

**Figure 12:**
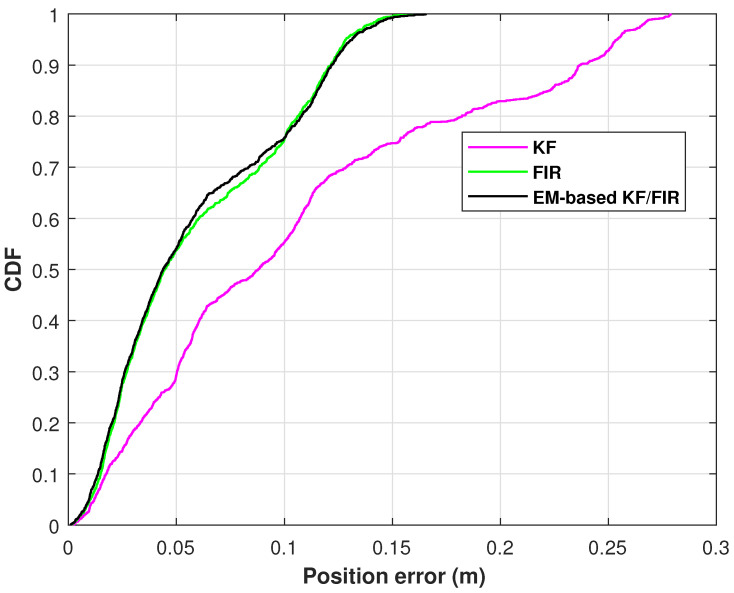
The position-error CDFs of the wrist measured by the KF, FIR filter, and EM-based KF/FIR filter in test 2.

Figure 12 illustrates the position-error CDF for the KF, FIR filter, and EM-based KF/FIR filter concerning wrist positions in test 2. The figure shows that at a probability of 0.9, the positioning error of KF is the largest and that of FIR is smaller than that of the KF; in addition, the proposed method aligns more closely with the KF solution.

This section substantiates the effectiveness of the proposed EM-based KF/FIR integrated filter in providing superior performance than the KF and FIR filters independently, successfully addressing the limitations inherent to both filters. It should be pointed out that the setting of the Q and R used in this work depend on all the sensors’ data in the test. Thus, we can obtain the accurate setting of the Q and R by using the sensor’s data. However, it should be pointed out that it is not easy to obtain in practice. Moreover, from the results mentioned above, we can see that not all Q and R are suitable for the KF algorithm, especially for the east direction.

### 4.4. Operation Time

In this section, the operation time of the filters used in the test will be compared. In this work, we employed the Lenovo Legion computer; its CPU is Intel(R) Core(TM) i7-10875H CPU @ 2.30 GHz, the RAM of the computer is 16 GB, and all the filters were run on Matlab R2017a. The running time of the KF, FIR filter, and EM-based KF/FIR filter in tests are listed in Table 6. From the table, we can see that the KF has the shortest running time, with a mean running time of 0.037 ms, and the proposed EM-based KF/FIR has the longest running time, with a mean running time of 7.954 ms. It should be pointed out that the sampling time is 33.33 ms, thus, although the running time of the proposed EM-based KF/FIR is longest when compared with the other filters, its value is smaller than the sampling time.

## 5. Conclusions

The increasing prevalence of motor function injuries presents substantial challenges for patients and their families. Consequently, the accurate execution of rehabilitation training has emerged as a critical research area. This study introduces an EM-based KF/FIR integrated filter for posture capture of human upper limbs, focusing on precise wrist and elbow position information. In this work, the wrist and elbow’s position have been considered. Thus, we employ their position and the velocity in East–North–Up frame as the state vector, and their positions measured by the IMUs are used as the measurements. The outputs from the two data fusion filters are then used to determine the posture of human upper limbs. In the proposed method, the filter performance is assessed using the *Mahalanobis* distance. When the performance of the KF is suboptimal, the EM-based KF is utilized to enhance performance. Subsequently, if the performance of the EM-based KF declines, the FIR filter is employed to increase localization accuracy. An EM-based KF/FIR integrated filter is used for the posture capture of human upper limbs. A real-world test was conducted to demonstrate the effectiveness of this approach. In the test, two IMUs provided INS-based wrist and elbow positions, while Kinect 2.0 was used to obtain reference values. The proposed EM-based KF/FIR integrated filter was compared with the traditional KF and FIR filter. The results indicated that the proposed EM-based KF/FIR integrated filter outperforms the conventional KF and FIR filter in localizing wrist and elbow positions.

## Figures and Tables

**Table 1:** Parameters of the IMUs involved in the test [29].

Parameter	Value
Max sampling frequency	100 Hz
Data transmission distance	100 m
Working voltage	4.2 V

**Table 2:** Kinect parameters set in the test [29].

Parameter	Value
Resolution of color image frames	1920×1080
Resolution of deep frames	512×424
Detectable range	0.5–4.5 m
Resolution of infrared image frames	512×484

**Table 3:** Parameters of the computer used in the test.

Parameter	Value
Processor	Intel(R) Core(TM) i7-10875H
Frequency	2.3 GHz
RAM	16 G

**Table 4:** RMSEs of the KF, FIR filter, and EM-based KF/FIR filter concerning elbow positions in test 1.

Methods	East Direction (m)	North Direction (m)	Up Direction (m)	Mean (m)
KF	0.178	0.011	0.010	0.066
FIR	0.086	0.014	0.014	0.038
EM-based KF/FIR	0.086	0.013	0.013	0.037

**Table 5:** RMSEs of the KF, FIR filter, and EM-based KF/FIR filter concerning wrist positions in test 1.

Methods	East Direction (m)	North Direction (m)	Up Direction (m)	Mean (m)
KF	0.036	0.020	0.020	0.025
FIR	0.069	0.014	0.014	0.032
EM-based KF/FIR	0.040	0.018	0.018	0.025

**Table 6:** The running time of the KF, FIR filter, and EM-based KF/FIR filter in tests.

Methods	Wrist (ms)	Elbow (ms)	Mean (m)
Sampling time	33.33	33.33	33.33
KF	0.035	0.038	0.037
FIR	0.124	0.350	0.237
EM-based KF/FIR	5.629	10.279	7.954

## Data Availability

Data are contained within the article.

## Abbreviations

The following abbreviations are used in this manuscript:

CDF	cumulative distribution function
ELM	extreme learning machine
EM	expectation–maximization
FIR	finite impulse response filter
GNSS	global navigation satellite system
INS	inertial navigation system
KF	Kalman filter
PDF	probability density function
RMSE	root mean square errors
MLE	maximum likelihood estimator
UWB	ultrawide band
